# Mirror Clock: A Strategy for Identifying Atomic Clock Frequency Jumps

**DOI:** 10.3390/s22228995

**Published:** 2022-11-21

**Authors:** Mochi Liu, Yu Chen, Qian Xu, Yuzhuo Wang, Yuan Gao, Aimin Zhang

**Affiliations:** 1 National Institute of Metrology (NIM), Beijing 100029, China; 2 China National Intellectual Property Administration, Beijing 100088, China

**Keywords:** atomic clock, frequency jump, random pursuit strategy, predictability

## Abstract

Atomic clock frequency jumps directly influence the accuracy and reliability of timekeeping systems. The necessary corrections are typically implemented by postprocessing mutual comparison data between multiple atomic clocks based on the overly strict assumption that these atomic clocks are independent of each other. This paper describes the concept of a mirror clock, which enables atomic clock frequency jumps to be identified in real time without any assumptions. By comparing whether the real measured data and a corresponding mirror clock prediction fall within a confidence interval determined by the uncertainty of past physical clock data, atomic clock frequency jumps can be effectively identified and corrected. The results of several experiments using three hydrogen masers verify that the precision and recall of simultaneous jump identification reach 96.41% and 73.49%, respectively.

## 1. Introduction

Atomic clocks play an important role in timing systems, including the physical realization of coordinated universal time (UTC) [1,2,3,4] and the time system of the global navigation satellite system (GNSS) [5,6,7]. Atomic clock frequency jumps sometimes occur in practical applications as a result of the principles on which the clocks are built and variations in ambient temperature and humidity [8,9]. To maintain the integrity of the timing system, it is important to quickly identify atomic clock frequency jumps [10,11,12,13].

Many studies have reported several methods for detecting atomic clock abnormalities [14,15,16,17,18]. However, it is difficult to evaluate the performance of atomic clocks in real time because there are no references with better frequency stability. In other words, if multiple atomic clocks with similar performance are synchronized with anomalies, then the anomalies cannot be identified through comparisons among these atomic clocks. Thus, in practice, the clock with the best frequency stability is used as the reference for detecting abnormalities. Of course, this raises the problem that the best-performing clock has abnormalities that cannot be monitored.

In theory, an ensemble of atomic clocks can be used to construct a collective reference timescale [10,19,20,21]. The frequency stability of this reference should outperform that of any single atomic clock in the ensemble. Although several studies have demonstrated that atomic clocks in the same laboratory could be affected by ambient influences [22,23], this approach is widely used to facilitate time keeping. The most stable and dependable timescale reference is UTC [24], but this can only evaluate the frequency stability of atomic clocks at time intervals of five days. This lag means that UTC falls well short of accurately evaluating the short-term performance of atomic clocks. In summary, the foundation for atomic clock evaluation is the establishment of a reliable, stable, real-time reference.

When combined with the actual demand for atomic timescale generation in time-keeping laboratories, the ideal reference must be available at the desired sample intervals. To overcome the disadvantages of local and physical references, an exploratory research concept that we refer to as “mirror clocks” is proposed herein. The fundamental idea behind these mirror clocks is the ensemble prediction model, which directly evaluates the measured frequency drift with a confidence interval generated based on the historical uncertainty of the real clock. This is used as a baseline for monitoring the abnormalities of the physical clock in real time.

## 2. Method

The mirror clock is inspired by the concept of “digital twins” [25] and learns the output characteristics of atomic clocks using parametric or nonparametric modeling to smooth, filter, and predict the atomic clock output signals. In this paper, an ensemble polynomial model is applied to the mirror clock concept to predict the atomic clock’s output. A confidence interval is output alongside the prediction to quantify the acceptable range of the real measured output. This enables the measurement accuracy to be evaluated based on a probability density function.

### 2.1. Mirror Clock Implementation Workflow

**Figure 1:**
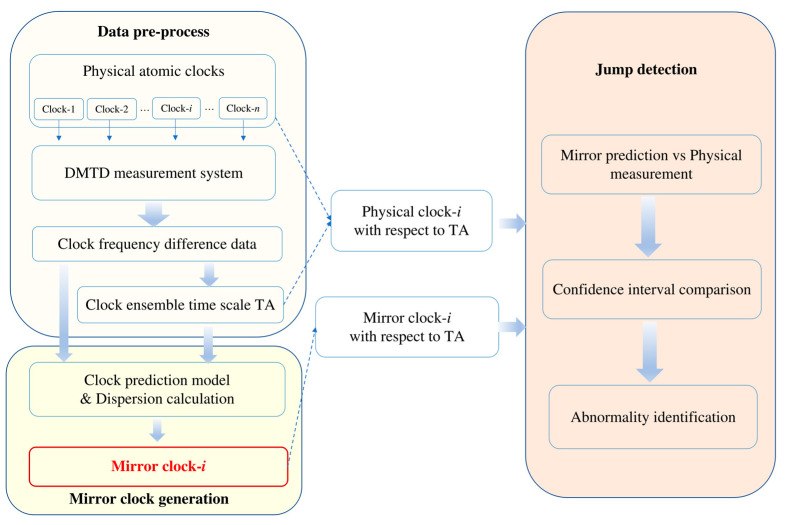
Mirror clock implementation flowchart.

The clock prediction model serves as the foundation for the mirror clock design and attempts to identify the trends in the clock frequency difference data. This allows the possible parameters of randomly sampled data to be estimated. Subsequently, the model enables future frequency difference data to be predicted based on a weighted sum approach. Therefore, mirror clocks are built using the ensemble algorithm based on past data from real atomic clocks. Mirror clocks anticipate the values and confidence intervals for one or more future frequency points and can be used to identify jump points as well as smoothing the clock data. This is helpful for improving the performance of the timescale in time-keeping systems. According to the key notions of mirror clocks, a technical solution is provided through the combination of three primary processing units: data preprocessing, mirror clock generation, and jump detection. Figure 1 depicts the specific implementation flowchart, and the following sections discuss each module in detail.

### 2.2. Data Preprocessing

The data preprocessing module collects clock frequency difference data from n clocks applying dual mixer time difference (DMTD) measurement system over a certain sampling time. The jumps in the data are discovered and corrected by postprocessing the clock difference data among the ensemble. The real-time clock ensemble timescale TA is then calculated using the classical clock ensemble approach with inverse variance weights. The Allan variance of the frequency data for each clock difference is calculated, and then the inverse of the variance is used as the ensemble weight, which is taken as the reference. The clock difference is used as the input for the next workflow module. Equations (1) and (2) describe these calculations:

(1) $$w_{i}=\frac{1/Var\left(h_{i}{}^{history},\;\tau \right)}{\sum_{i=1}^{n}1/Var\left(h_{i}{}^{history},\;\tau \right)}$$

(2) $$\mathrm{TA}=\sum_{i=1}^{n}w_{i}\times h_{i}\;$$

where $Var\left(\cdot ,\;\tau \right)$ denotes the Allan variance at averaging time τ; Both $h_{i}{}^{history}\;$(*i* = 1, 2, …, *n*) and $h_{i}$ (*i* = 1, 2, …, *n*) denote the respective clock readings with respect to the reference; $h_{i}{}^{history}$ represents the historical data of the ith clock of fixed length. While $Var\left(h_{i}{}^{history},\;\tau \right)$ reflects the historical stability of the ith clock at averaging time τ. For the selected clocks in mirror clock generation and jump detection, $Var\left(h_{i}{}^{history},\;\tau \right)$ takes a fixed value and does not change by time. $h_{i}$ represents the clock data immediately follows the historical data and is used for mirror clock generation and jump detection. TA denotes the clock ensemble timescale and is used as the reference. The clock difference $h_{i}-\mathrm{TA}$ is then passed to mirror clock generation.

### 2.3. Mirror Clock Generation

The mirror clock generation module first calculates the standard deviation $\sigma_{i}$ for the clock with respect to the reference as the clock data dispersion. The data for each clock difference are then input to the mirror clock prediction algorithm. The random pursuit strategy (RPS) [26,27] is used for drift learning due to its stronger ability to suppress the influence of frequency jump on clock prediction. Theoretically, other clock prediction methods, such as KF, are also candidates to replace RPS in the process of mirror clock generation.

Each clock difference sequence with respect to the ensemble timescale TA in the sample window is divided into k random subsequences, and k regression models are fitted for each of these subsequences by fixing the starting point and setting the number of sampling points to$\;m_{2}$. An ensemble of these regressions is used to obtain the final prediction. While moving the sampling window, the algorithm continually anticipates one point in the future. Hence, the model runs in real time. Equations (3)–(5) describe the detailed calculations:

(3) $${\hat{h}}_{i}^{j}=reg_{j}\left(h_{i}^{j}\right)$$

(4) $$w_{i}^{j}=\frac{err_{j}\left(h_{i}^{-j}\right)}{\sum_{j=1}^{k}err_{j}\left(h_{i}^{-j}\right)}$$

(5) $$h_{i}^{\mathrm{mirror}}={\hat{h}}_{i}=\sum_{j=1}^{k}w_{i}^{j}\times {\hat{h}}_{i}^{j}$$

where $h_{i}^{j},\;\left(i=1,\;2,\;\ldots ,\;n,\;\;j=1,\;2,\ldots ,k\right)$ represents the jth subsequence obtained by randomly grouping the sampled sequences of the ith continuous clock reading; $h_{i}^{-j}$ represents the subsequence of the ith continuous clock data after removing the jth subsequence; $reg_{j}\left(h_{i}^{j}\right)$ denotes the prediction value obtained by regression using the jth subsequence; and $err_{j}\left(h_{i}^{-j}\right)$ denotes the sum of squared errors of the prediction using the regression model learned from the subsequence of the ith continuous clock data after removing the jth subsequence. ${\hat{h}}_{i}$ is the predicted value of the ith clock using the RPS algorithm, which is also the reading of the mirror clock for the *i*th clock.

### 2.4. Jump Detection

A confidence interval is provided for the predicted values of the mirror clock. This interval, ${\hat{h}}_{rps}^{i}\pm rps\_bound\times \sigma_{i}$, combines the predicted value of the RPS method and the standard deviation of prediction errors obtained by RPS method for the past clock difference data. The physical measured data from the real clock are regarded as potential jump points if they are located outside of the confidence interval. The system is adjusted, and the clocks’ stability and performance are periodically assessed.

Obviously, the abnormality detection method proposed herein is achieved through comparisons between each physical clock and its own mirror clock. At the same time, to ensure that the references for the physical and mirror clocks are sufficiently reliable, they are both derived from the ensemble timescale TA.

## 3. Experiment Preparation

**Figure 2:**
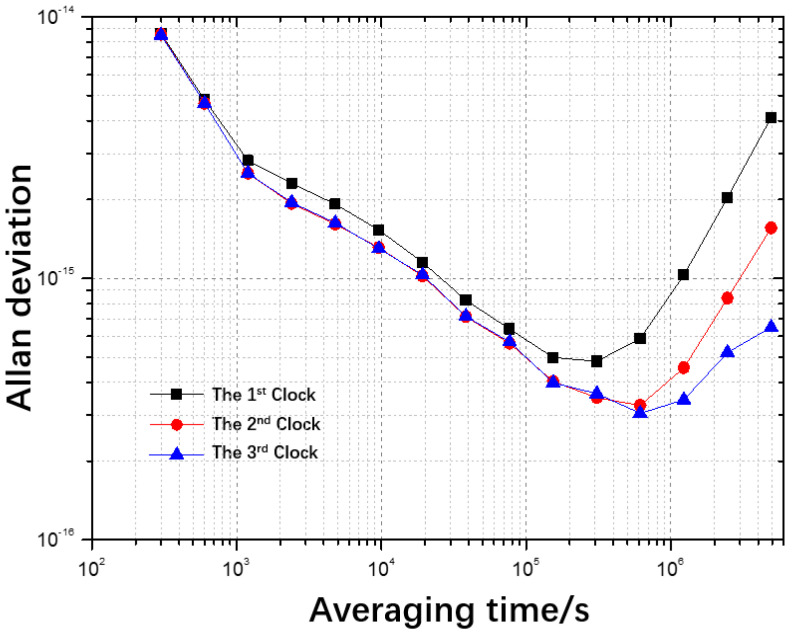
Allan deviation of three hydrogen masers with respect to UTC(NIM).

In a practical experiment, 40,000 sampling points were collected over a minimum sampling interval of 5 min from three hydrogen masers maintained at the National Institute of Metrology (NIM) in China (Beijing). The clock difference data were obtained with respect to UTC(NIM), i.e., the physical realization of UTC. The frequency stability curves are displayed in Figure 2.

To test the mirror clocks’ functionality and the proposed algorithm’s accuracy, experiments were designed to detect jumps under various conditions. There are various scenes of real clock jumps. A single clock jump is the simplest and most common scenario. In a clock set, two clocks may jump non-simultaneously. In this case, we can analyze for each clock individually and identify the jumps. While if two or more clocks jump in the same direction and simultaneously, it is often difficult to probe the jumps, and it may even be possible to conclude that other clocks are jumping. Hence, to better simulate and deal with real clock jumps, two scenarios are assessed as follows: ‘a single clock jump’ and ‘two simultaneous clock jumps in the same direction’.

### 3.1. Single Clock Jump

A single clock jump is quite common. For each clock, 2000 out of the 40,000 sampling points were selected by random sampling. The jumps were injected with a uniform distribution $jump_{i}\sim U\left(\sigma_{i}^{jump}\times lower,{\;\sigma }_{i}^{jump}\times upper\right)$, where lower=3.0, upper=7.0. $jump_{i}^{s}$ or $-jump_{i}^{s}$ is then added to the sth selected sample point of the ith clock. Here, $\sigma_{i}^{jump}$ is the standard deviation of 40,000 sample points from the ith clock respective to its reference, representing the dispersion of these sample points. In other words, it is calculated on $h_{i}\;$but not on the historical sample points $h_{i}{}^{history}\;$in the preprocessing phase.

### 3.2. Two Simultaneous Clock Jumps in the Same Direction

In this scenario, two clocks simultaneously jump in the same direction. The clock difference data for two of the three clocks were handled by randomly selecting 2000 sample points from the total of 40,000 sampling points. The jumps were injected with a uniform distribution $jump_{i}\sim U\left(\sigma_{i}^{jump}\times lower,{\;\sigma }_{i}^{jump}\times upper\right)$. By adding $jump_{i}^{s}$ or $-jump_{i}^{s}$ to the sth selected sample point in the same direction to both clocks. The definition of $\sigma_{i}^{jump}$ is the same as for the single clock jump.

### 3.3. Sliding Prediction Model with RPS Strategy

The clock difference sequence was used in the sliding prediction model based on the RPS method, as described in Section 2.3. The model enables the sample points in a sliding window to be predicted in real time. Considering the noise characteristics of the clock frequency and the computational cost, the RPS hyperparameters were set to rp_win_size=288 and k=4. Here, rp_win_size is the number of sampling points in the sliding window and k represents the number of sub-sequences of RPS. The model was tested with rps_bound = 2, 3, and 4 to fine-tune the prediction model.

## 4. Evaluating Clock Jump Detection Capability

The performance of jump point identification was measured by counting the valid jump detections over 10 experiments. These data were then used to calculate the average and standard deviation of the precision and the average and standard deviation of the recall.

The criterion for determining a valid jump is whether the value after adding the jump is located outside of $\mu_{i}\pm 3\times \sigma_{i}^{jump}$, where $\mu_{i}$ represents the mean value of the ith clock in a rolling window and $\sigma_{i}^{jump}$ represents the dispersion of the ith clock calculated with the 40,000 sample points. The precision and recall of valid jump detection illustrate the capability of our model to detect outliers. Equations (6) and (7) were used to compute the precision and recall, respectively:

(6) $$\mathrm{precison}=\frac{\mathrm{TP}}{\mathrm{TP}+\;\mathrm{FP}}$$

(7) $$\mathrm{recall}=\frac{\mathrm{TP}}{\mathrm{TP}+\mathrm{FN}}$$

where TP denotes true positive, which means the proposed method successfully detects valid jumps, FP denotes false positive, which means the proposed method detects normal operation as a valid jump, and FN denotes false negative, which means the proposed method does not detect a valid jump. Under the optimal hyperparameters, the statistical results of valid jump detection for the three clocks are listed in Table 1.

**Figure 3:**
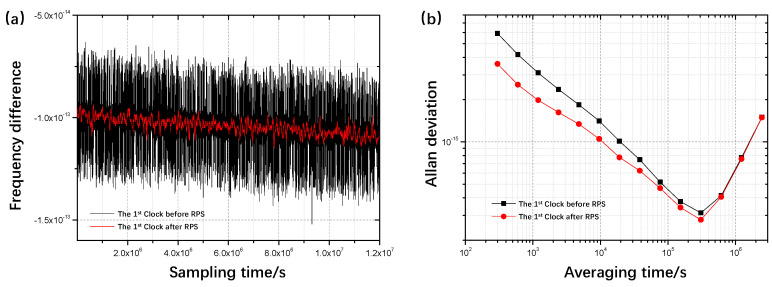
First hydrogen maser and its mirror clock (**a**) and the stability curves (**b**) of this hydrogen maser with jump and its mirror clock in the case of a single clock jump.

Figure 3a shows the frequency difference curves of the first hydrogen maser with the jump added and its mirror clock with respect to the same reference. Obviously, the mirror clock (red line) has a smaller frequency range than the first hydrogen maser, and the drift in the mirror clock frequency can be clearly seen.

The jump point can be corrected in the desired sample interval once the model detects an error in the atomic clock. The corrected atomic clock can be created by swapping the jump for the expected value of the mirror clock. The stability curves of the first atomic clock before and after correction are depicted in Figure 3b. In terms of short-range stability, the corrected atomic clock (red line) performs better than the hydrogen maser. This advantage progressively diminishes over the longer term because the effect of the jump on the stability of the full clock ensemble is mostly evident over short periods of time. The mirror clock effectively addresses the instability that the jump has introduced.

### Detection of Two Simultaneous Clock Jumps

With two clocks jumping in the same direction at the same time, jump detection occurs when the model identifies the jump points simultaneously for both clocks. This means that, when the model predicts a jump for the first clock, it must also identify a jump for the second clock.

In the case of the first and second clocks jumping, under the optimal hyperparameters, the average recall of model identification is 96.41% ± 1.43% and the average precision is 73.49% ± 2.31%. These performance metrics were calculated for all valid jumps and for each single clock. The performance evaluation of the detection of simultaneous valid jumps in two clocks for different values of rps_bound is presented in Table 2.

In the case of the two clocks jumping, the frequency curves of any hydrogen maser with respect to the jump and its mirror clock are consistent with the single clock jump scenario. The rectified atomic clock performs similarly to the hydrogen maser in terms of long- and short-range stability. This outcome is very similar to the circumstance of a single clock jump. The results demonstrate that, even in the case of many clocks jumping in the same direction, the proposed model retains good detection and correction capabilities.

## 5. Discussion

Section 3 described how the predicted values could be obtained through the RPS strategy for a hydrogen maser. Here, standard deviation is used to characterize the dispersion of clock prediction errors at short-term time. When considering to long-term prediction, Allan deviation could be more suitable due to the change of dominant clock noise. The robust prediction model of RPS means that the proposed mirror clock method is also suited to other kinds of clocks. The selection of the hyperparameters is an important part of the optimal RPS model. Table 2 presents results for different values of rps_bound, with the optimal values of the other hyperparameters being determined experimentally.

The results in Table 2 suggest that the precision and the recall of jump detection are well balanced when *rps_bound* is set to 3. This is consistent with the *three-sigma* principle given by statistical theory. However, this does not prevent *rps_bound* from being adjusted to obtain higher precision or higher recall in particular applications. Here, rps_bound is an effective regulation parameter that can be used to determine the tolerance of the system to jumps.

The solution to simultaneous jump detection has been examined in detail. Although the probability of simultaneous jumps is not high relative to single jumps, no reasonable solution for the detection of such jumps has previously been reported. As described above, the mirror clock becomes its own reference for each clock, so we can detect the behavior of the mirror clock independently. Therefore, simultaneous valid jump detection becomes a simple combination of detecting single valid jumps.

Analysis of all detection results indicates that the main reason for the failure to detect jumps is the limitation of determining a confidence interval via the historical uncertainty of the real clock. This assumes that the noise characteristics of the detected real-time data are consistent with the noise characteristics of the historical data, which are increasingly biased over time. Additionally, the noise characteristics of the historical data are inherently complex, and analyzing their variance alone, while simplifying the calculation, may result in a loss of accuracy.

## 6. Conclusions

Mirror clocks provide a novel means of detecting jumps in real time, while reducing the cost of the clock ensemble hardware. Co-directional jumps in multiple clocks can be detected. The predicted data from the mirror clock provide a baseline for the real measured data, and the confidence interval based on the historical uncertainty of the real clock gives an acceptable range for the difference between the real measured data and the predicted data. The experimental results presented in this paper confirm that the proposed method is reliable and effective.

## Figures and Tables

**Table 1:** Statistical result of valid jump detection.

Metrics	Recall	Precision
1st clock	99.23% ± 0.54%	82.97% ± 1.02%
2nd clock	87.01% ± 1.34%	80.72% ± 1.68%
3rd clock	95.41% ± 0.45%	71.87% ± 2.57%.

**Table 2:** Statistical results for the detection of two clocks jumping.

	Parameter	rps_Bound				
Metrics		2	2.5	3	3.5	4
Number of valid jumps added to 1st clock		1289 ± 24	1283 ± 34	1285 ± 26	1290 ± 35	1277 ± 30
Number of valid jumps added to 2nd clock		1019 ± 21	1014 ± 29	1017 ± 16	1023 ± 30	1009 ± 38
Recall of valid jump detection for 1st clock		100% ± 0%	100% ± 0%	**99.74% ± 0.12%**	95.83% ± 0.98%	75.39% ± 1.18%
Precision of valid jump detection for 1st clock		69.87% ± 0.74%	72.28% ± 1.07%	**81.09% ± 2.53%**	94.15% ± 1.78%	99.03% ± 0.39%
Recall of valid jump detection for 2nd clock		100% ± 0%	99.88% ± 0.07%	**88.30% ± 1.30%**	63.87% ± 2.42%	19.77% ± 2.43%
Precision of valid jump detection for 2nd clock		39.33% ± 1.08%	52.34% ± 0.81%	**73.88% ± 1.91%**	97.86% ± 1.47%	98.73% ± 0.26%
Recall of intercept valid jump detection for two clocks		100% ± 0%	99.88% ± 0.08%	**96.41% ± 1.43%**	62.66% ± 2.51%	6.91% ± 0.70%
Precision of intercept valid jump detection for two clocks		38.60% ± 1.25%	51.52% ± 1.31%	**73.49% ± 2.31%**	98.59% ± 0.25%	98.63% ± 0.12%

## Data Availability

Data available on request from the authors.
